# Re-evaluation of Significance and the Implications of Placebo Effect in Antidepressant Therapy

**DOI:** 10.3389/fpsyt.2019.00143

**Published:** 2019-03-19

**Authors:** Marko Curkovic, Andro Kosec, Aleksandar Savic

**Affiliations:** ^1^Department for Diagnostics and Intensive Care, University Psychiatric Hospital Vrapce, Zagreb, Croatia; ^2^Department of Otorhinolaryngology and Head and Neck Surgery, University Hospital Center Sestre Milosrdnice, Zagreb, Croatia; ^3^Department of Psychiatry, University of Zagreb School of Medicine, Zagreb, Croatia

**Keywords:** depression, antidepressants, placebo, placebo effect, efficacy, randomized controlled trials

## Introduction

Placebo was conceived as an epistemological tool to control for incidental factors that could influence investigated outcomes (1, 2). As a phenomenon, placebo has been conceptualized in many different ways, both theoretically and practically, although a generally accepted definition is still to be devised (3). In clinical research setting it is, however, helpful to distinguish placebo response from placebo effect. Placebo response is considered to be the composite change observed in individuals after administration of placebo, consisting of different aspects, such as natural course of the disease and methodological artifacts, as well as the placebo effect itself (4, 5). Placebo effect would therefore be the change observed in individuals after controlling for natural course of the disorder, methodological aspects, and the effect linked to treatment-specific features (i.e., antidepressant verum) (1). In other words, placebo responses may or may not include placebo effect as genuine psychobiosocial effect that is usually attributed to various features of treatment situation and contexts (5). Recent findings consistently show a modest average effect (a mean effect size of *d* = 0.30) of antidepressants above placebo in short-term treatment of adult depression (6, 7). This could be interpreted as strong evidence of a modest effect. Whether this effect on outcome measures, that are rather limited and subjective, is clinically meaningful remains an open question (8). Issues of true long-term efficacy, safety and cost-effectiveness of antidepressant drugs still loom over the horizon (8–11). Still, these recent findings pose interesting questions, since similarly consistent findings have been published showing a large and highly variable placebo effect in studies aiming to prove true efficacy of antidepressants (12, 13). In other words, participants in antidepressant studies that are receiving, at least from a theoretical point, a supposedly inherently neutral intervention (one that should be lacking known, relevant, and specific features), show substantial and consistent improvement across different study designs and contexts.

## Context

Double blind randomized placebo-controlled trials (DBRPCT) have long been considered to be the golden standard for determining true efficacy of an intervention. As such, DBRPCTs are based on the logic that reflects the basic premises of scientific epistemology: it allows a certain degree of control over factors that could influence outcomes of interest, but at the current point is not a subject of scientific inquiry (3). Randomization, blinding, and placebo control groups allow for probabilistic balancing of these unspecific factors, and prevent intentional or accidental influence from study participants and investigators (14). Consequently, it is assumed that true effect of intervention could be extracted based on the “additivity assumption”—true effect is one that is present only within the last remaining uncontrolled therapeutic features, and therefore attributed to the intervention being investigated. Such a design, created for a very specific purpose, has its shortcomings that have been widely discussed [for more comprehensive discussion consider (14) and related commentaries]. In order for a DBRPCT to be internally valid (able to fulfill its explanatory purpose), a certain degree of deviation from external validity is required, causing loss of similarity to the targeted model—clinical practice (14, 15). Placebo in DBRPCTs was initially conceived as a procedure that allows blinding, removing the influences of study participants and investigators. It seems, however, that placebo consistently influences outcomes above and beyond its anticipated boundaries (1, 16, 17). Although we aim to constrain “human factor,” certain aspects of human nature evade such attempts. Human subjects, inherently vulnerable because of the nature of their medical condition, are systematically and consistently reacting to a more or less specific set of internal and external cues, creating a “genuine” placebo effect. While placebo effect may be a valuable and legitimate object of research, one should be careful not to overgeneralize this term, since a tendency to erroneously characterize everything and anything as a placebo effect can be seen [for more detailed discussion consider (18)]. In other words, as previously mentioned, genuine placebo effect should be distinguished from methodological artifacts that exert certain influence on outcomes, such as natural course of the investigated condition, spontaneous variation in symptoms, and various sources of research bias (2, 18, 19). It seems that genuine placebo effect exercises a greater influence in its own right than any of above mentioned factors, and as such is neither inert nor unspecific. It is responsible for physical changes and effects in individuals that are specific and somewhat related to the investigated condition and/or effects of “true” treatment (1, 2, 16, 19, 20). The placebo effect is considered to be an adaptive process that emerges from contextual and individual features within a treatment situation, and as such is driven by underlying biological, psychological and social components that are not mutually exclusive [for further details consider: (1, 21)]. As such, the placebo effect may contradict the additivity assumption, influencing outcomes conjointly or even independently from the investigated intervention (1). Therefore, an “interactive” assumption has been proposed, acknowledging that underlying mechanisms that yield a therapeutic response interact in a complex manner (1, 22).

## Recent Findings

Findings suggest that placebo effect in antidepressant trials is a genuine entity, and as such may be distinguished from methodological artifacts that are also exhibiting a substantial influence on outcomes (23–25). While recent findings suggest that antidepressants show therapeutic efficacy and effectiveness, it seems that placebo effect may be one of the key driving forces of their effect. Moreover, it has been suggested that as much as 88% of antidepressants efficacy could be attributed to the placebo effect (8). In other words, antidepressants would in that case have little additional specific effect beyond the placebo effect. Furthermore, recent analyses found that a subset of 17% of individuals with depression could exhibit “clinically significant advances” with placebo relative to antidepressants (26). Similarly, earlier findings suggested that 20% of individuals with depression could have a worse disease trajectory with antidepressant than with placebo therapy (27).

Moderators and mediators of placebo and antidepressant effects have been thoroughly investigated and reviewed [more thoroughly discussed in: (28, 29)]. Unbalanced studies group randomization and effect modulation by baseline severity have been previously singled out as most consistent and robust findings. It may seem intuitive that baseline severity of depression influences responses to any given intervention, and it has long been argued that as depression is more severe, placebo effects are less prominent, while response rates to antidepressants remain stable (28, 30). This concept was recently dismissed, as antidepressants or placebo intervention seems to be equally (in)effective across the whole depression severity spectrum (31, 32). Interestingly, recent findings even suggest that placebo response rates seem to be similar in persistent depressive disorder (defined as all forms of depressive conditions that persist for at least 2 years) compared to episodic depression (33). The probability of receiving placebo (unbalanced group randomization) has been repeatedly and firmly correlated with the antidepressants' response (12, 29, 34). This relationship has a linear gradual effect, with efficacy of antidepressants increasing as we move from greater toward lower probability of receiving placebo. So, antidepressant response rates are significantly higher in comparator trials than in DBRPCT. This finding is usually interpreted as implicit evidence that both placebo and treatment effects could be based on patient expectations (that could obviously be positive and/or negative) (12). Nonetheless, it has been shown that expectations (conceptualized as perceived treatment assignment) significantly change during studies while retaining their relative predictive power (35). What participants believe may be more important than what they actually receive as an intervention, making a false but sincerely held belief more important that actual intervention. Some advancement has been made in predicting antidepressant and placebo responses and/or responsiveness in research and clinical practice. Although certain neurobiological features, clinical and socio-demographic characteristics of patients have been highlighted as possible outcome predictors, low sensitivity and high intra- and inter-individual variability remain an issue (26, 36–38). Placebo responsiveness, and to lesser account antidepressant responsiveness remain highly and complexly variable on all levels.

## Does Placebo Effect Have an Effect Above and Beyond That of Antidepressants?

Many questions are still unanswered regarding characteristics, mechanisms and definition of the placebo response and effect. Line of research dealing with those issues could be referred to as “placebo explanatory research,” and depression and psychiatry disorders in general could be seen as particularly fertile ground for these inquiries (1, 2, 16, 24, 25, 29). For example, open-label placebo administration with full disclosure, seems to yield similar antidepressant therapeutic effects as the traditionally administered ones (39). On the other hand, antidepressants compared with active placebos that imitate some side-effects showed no significant advantage (40). Thus, expectation related placebo effects may be driven by unblinding properties of side-effects, and further diminishing antidepressants' signaling potential (extricating true efficacy).

We consider “antidepressant explanatory research” as one being primarily oriented toward proving true antidepressants efficacy. Within this approach, it has been recently argued that the placebo control group should be completely omitted, as diverse variability of placebo effects seems to undermine internal validity making studies fundamentally invalid and uninterpretable (7, 12). Proponents of keeping the placebo control group, propose methodological and analytical approaches that aim to control and manage placebo effects [further details may be found in: (1, 41, 42)]. One of the underlying assumptions is that “placebo responders” influence outcomes blurring the antidepressants' signaling potential. Hence, different methods, such as placebo run-in phase could be applied in order to eliminate these obstacles. This approach should be considered ethically and methodologically erroneous as there is no evidence that such a stable trait exists. Just the opposite, it seems that placebo responsiveness emerges from complex interrelationship between stable and situational traits [recently elaborated in: (19)]. Furthermore, it could be argued that reduction of placebo responsiveness will further reduce antidepressants responsiveness (25). Similar logic is applied within the approach of risk modeling where “risk participants” (disproportionately contributing to the outcome) and/or “non-responders” (not prone to react regardless of assigned intervention) are further dealt in identify and mitigate manner (1, 42). All of these strategies may be considered pragmatic, as there is great pressure to reduce ineffectual research. There are other strategies that tackle different possible sources of error by manipulating study context, design, conduct and analysis with primary aim to enhance studies' internal validity, antidepressants' signal detection potential and yield more historically reliable response rates (1, 2, 16, 20, 25, 28, 41–43). However, these strategies tend to increase internal validity at the expense of external validity, and as such seem more like a harm reduction strategies than as true advancement of our understanding of the complex underlying phenomena (14, 18, 25, 44, 45). Following this line of argumentation, solutions could include introduction of an independent study investigator and the concept of “cold standardization”—a virtual, computer driven standardized recruitment, admission of interventions and assessment of study participants. Such an approach would have potential to eliminate some features of intrapersonal healing that has been singled out as possibly one of the major contributors to the placebo effect, tackle widespread issue of inadequate blinding and other sources of investigator or study-staff related biases (14). Although such (still hypothetical) computerized study investigator could standardize study recruitment, administration and assessment procedures, it would not be resistant to other sources of bias. One could even imagine that participants' expectations in such a setting would change in previously unimaginable directions (either by certain therapeutic potential of this interaction or properties of interventions itself, such as side effect profile). Although here being used as extreme argument on how one could possibly further strengthen studies internal validity, such an approach could be also used in order to distinguish specific features underlying placebo and/or therapeutic effects (serving a more pragmatic purpose).

Alternatively, “antidepressant pragmatic research” would steer toward comprehending complex interactions of specific and/or unspecific features that are contributing to a therapeutic effect. We should not try to simply manage placebo effects, but direct our attention to its understanding through rigorous initial planning, assessment, reporting and sharing of all data possibly linked to the therapeutic response as well as non-response (1, 17, 19, 20, 43, 44). In other words disentangling of the placebo enigma seems to carry the potential of being the royal road to answering presumably the most important question at hand: which elements of the intervention, and in what proportion, are the ones relieving the suffering? In that sense, inclusion of an additional study arm in which the primary aim is to reach the maximum possible efficiency through any means necessary could be labeled as “warm standardization” (25). Again, different means could be used for that purpose, for example harnessing and maximizing expectations or even including additional specific interventions (such as some form of specific psychotherapy—being previously conceived as inherently expectation modulatory treatment) (46). Finally, as placebo is a relational phenomenon that significantly differs from context to context, all known moderators and mediators of placebo effect (from its physical characteristics to informed consent process) should be rigorously reported (1, 2, 16, 17, 43). Factors that affect treatment outcomes need to be evidenced, extrapolated, weighted, agglomerated, and discussed having in mind that acquiring scientifically grounded knowledge is an iterative, cumulative process. Currently, novel analytical tool, such as computational methods allow us to amplify robustness of other data rich sources, such as electronic health records, while searching for the structures of causality that could be more rooted in real world estimates of certain interventions safety, efficacy and effectiveness (14, 45, 47–49).

## Author Contributions

MC provided and constructed initial idea of the manuscript. MC, AS, and AK co-authored and edited the manuscript.

### Conflict of Interest Statement

AS has received lecture honoraria from Janssen, Lundbeck, Eli Lilly, Pfizer, Pliva, Krka, Belupo, and participated in clinical trials (sub-investigator/rater) for Otsuka, Affiris, Eli Lilly. The remaining authors declare that the research was conducted in the absence of any commercial or financial relationships that could be construed as a potential conflict of interest.
